# Supplementary material for: Relationship between Cardiac Acoustic Biomarkers and Pulmonary Artery Pressure in Patients with Heart Failure
Source: J Clin Med. 2022 Oct 28;11(21):6373. doi: 10.3390/jcm11216373 (PMC9655038; doi:10.3390/jcm11216373)
Supplement: Supplementary file 1 [file jcm-11-06373-s001.zip › jcm-1957082-supplementary.pdf]

Supplementary Materials

Figure S1. Scatter plots of mean PAP and S3 CABs.

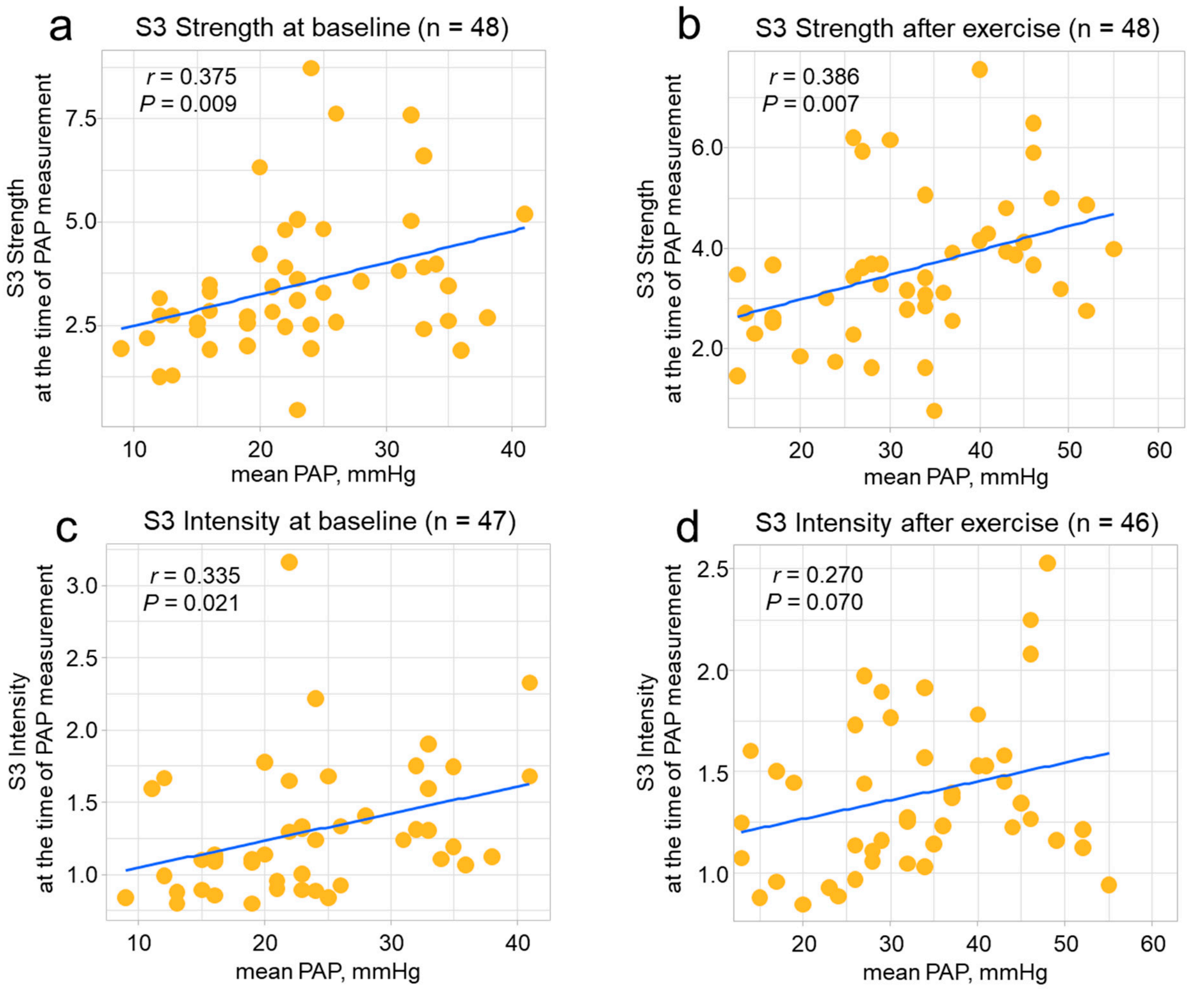

Scatter plots of mean PAP at baseline (a and c) and after exercise (b and d) and concurrently recorded S3 Strength (a and b) and S3 Intensity (c and d) are shown. The blue lines represent regression lines for each parameter correlation.

Abbreviations: CABs, cardiac acoustic biomarkers; PAP, pulmonary artery pressure; S3, third heart sound.

Table S1. Correlation between absolute values of PCWP and CABs.

	Mean PCWP			
	at baseline		after exercise	
	n	*r* (95% confidence interval)	n	*r* (95% confidence interval)
HR, bpm	49	0.201 (−0.085, 0.456)	48	0.319 (0.038, 0.553)*
S1 Intensity, mV	49	−0.127 (−0.395, 0.159)	48	−0.106 (−0.379, 0.183)
S1 Width, msec	49	−0.245 (−0.492, 0.039)	48	−0.344 (−0.572, −0.067)*
S1 Complexity	49	−0.274 (−0.515, 0.008)	48	−0.260 (−0.507, 0.026)
S2 Intensity, mV	49	0.220 (−0.065, 0.472)	48	0.220 (−0.069, 0.474)
S2 Width, msec	49	0.308 (0.030, 0.543)*	48	0.336 (0.058, 0.566)*
S2 Complexity	49	0.355 (0.082, 0.578)*	48	0.040 (−0.247, 0.320)
S3 Strength	49	0.404 (0.138, 0.615)*	48	0.353 (0.076, 0.579)*
S3 Intensity, mV	47	0.389 (0.114, 0.608)*	47	0.076 (−0.215, 0.356)
S4 Strength	34	−0.208 (−0.510, 0.140)	33	−0.070 (−0.404, 0.280)
S4 Intensity, mV	34	−0.026 (−0.361, 0.315)	32	−0.065 (−0.405, 0.290)
S2/S1 Intensity	49	0.211 (−0.075, 0.465)	48	0.324 (0.044, 0.557)*
S2/S1 Complexity	49	0.187 (−0.099, 0.445)	48	0.156 (−0.134, 0.422)

*: $P < 0.05$, r: correlation coefficient.

Abbreviations: CABs, cardiac acoustic biomarkers; PCWP, pulmonary capillary wedge pressure; HR, heart rate; S1, first heart sound; S2, second heart sound; S3, third heart sound; S4, fourth heart sound.

Table S2. Correlation between changes from baseline to post-exercise in PCWP and CABs.

	Mean PCWP	
	n	*r* (95% confidence interval)
HR, bpm	48	0.240 (−0.048, 0.491)
S1 Intensity, mV	48	−0.004 (−0.287, 0.281)
S1 Width, msec	48	−0.154 (−0.42, 0.136)
S1 Complexity	48	−0.033 (−0.314, 0.253)
S2 Intensity, mV	48	−0.099 (−0.372, 0.191)
S2 Width, msec	48	0.096 (−0.193, 0.37)
S2 Complexity	48	0.065 (−0.223, 0.343)
S3 Strength	48	0.170 (−0.120, 0.433)
S3 Intensity, mV	45	−0.187 (−0.455, 0.113)
S4 Strength	32	−0.048 (−0.390, 0.306)
S4 Intensity, mV	31	0.217 (−0.149, 0.53)
S2/S1 Intensity	48	−0.092 (−0.367, 0.197)
S2/S1 Complexity	48	0.136 (−0.154, 0.404)

r: correlation coefficient.

Abbreviations: CABs, cardiac acoustic biomarkers; PCWP, pulmonary capillary wedge pressure; HR, heart rate; S1, first heart sound; S2, second heart sound; S3, third heart sound; S4, fourth heart sound.

Table S3. Comparison of background characteristics with S3 Strength response to exercise-induced PCWP change.

		Overall	S3 Strength response		*P* value
			Decreasing (n = 25)	Increasing (n = 23)	
Age, number (%)					
(median)	< 69 years	23 (47.9)	13 (52.0)	10 (56.5)	0.578
	≥ 69 years	25 (52.1)	12 (48.0)	13 (43.5)	
BMI, number (%)					
(median)	< 24.0 kg/m^2	24 (50.0)	9 (36.0)	15 (65.2)	0.082
	≥ 24.0 kg/m^2	24 (50.0)	16 (64.0)	8 (34.8)	
eGFR, number (%)					
	< 60.0 mL/min/1.73m^2	31 (64.6)	18 (72.0)	13 (56.5)	0.367
	≥ 60.0 mL/min/1.73m^2	17 (35.4)	7 (28.0)	10 (43.5)	
NT-proBNP, number (%)					
(median)	< 1353 pg/mL	15 (51.7)	11 (73.3)	4 (28.6)	0.027
	≥ 1353 pg/mL	14 (48.3)	4 (26.7)	10 (71.4)	
Atrial fibrillation, number (%)					
	Yes	19 (39.6)	10 (40.0)	9 (39.1)	1.000
	No	29 (60.4)	15 (60.0)	14 (60.9)	
Diabetes mellitus, number (%)					
	Yes	16 (33.3)	12 (48.0)	4 (17.4)	0.034
	No	32 (66.7)	13 (52.0)	19 (82.6)	
Hypertension, number (%)					
	Yes	28 (58.3)	18 (72.0)	10 (43.5)	0.078
	No	20 (41.7)	7 (28.0)	13 (56.5)	
β-blocker use, number (%)					
	Yes	33 (68.8)	18 (72.0)	15 (65.2)	0.757
	No	15 (31.2)	7 (28.0)	8 (34.8)	
LVEF, number (%)					
	< 40%	31 (64.6)	15 (60.0)	16 (69.6)	0.490
	40-49%	7 (14.6)	3 (12.0)	4 (17.4)	
	≥ 50%	10 (20.8)	7 (28.0)	3 (13.0)	

E/e′, number (%)					
	< 14	30 (62.5)	20 (80)	10 (43.5)	0.016
	≥ 14	18 (37.5)	5 (20)	13 (56.5)	
Cardiac index, number (%)					
	< 2.2 mL/m^2	26 (54.2)	11 (44.0)	15 (65.2)	0.161
	≥ 2.2 mL/m^2	22 (45.8)	14 (56.0)	8 (34.8)	
Mean PCWP, number (%)					
	< 15 mmHg	25 (52.1)	16 (64.0)	9 (39.1)	0.148
	≥ 15 mmHg	23 (47.9)	9 (36.0)	14 (60.9)	
Mean PAP, number (%)					
	≤ 20 mmHg	18 (38.3)	11 (44)	7 (31.8)	0.549
	> 20 mmHg	29 (61.7)	14 (56)	15 (68.2)	
PH, number (%)					
	Yes (Ipc-PH or Cpc-PH)	22 (48.9)	15 (62.5)	13 (61.9)	0.139
	No	23 (51.1)	8 (37.5)	8 (38.1)	

Abbreviations: PCWP, pulmonary capillary wedge pressure; S3, third heart sound; BMI, body mass index; eGFR, estimated glomerular filtration rate; NT-proBNP, N-terminal pro-B-type natriuretic peptide; LVEF, left ventricular ejection fraction; PAP, pulmonary artery pressure; Ipc, isolated post-capillary; Cpc, combined post- and pre-capillary; PH, pulmonary hypertension.

Table S4. Comparison of background characteristics with S2 Width response to exercise-induced PAP change

		Overall	S2 Width response		*P* value
			Decreasing (n = 26)	Increasing (n = 22)	
Age, number (%)					
(median)	< 69 years	24 (50.0)	10 (38.5)	14 (63.6)	0.147
	≥ 69 years	24 (50.0)	16 (61.5)	8 (36.4)	
BMI, number (%)					
(median)	< 24.0 kg/m^2	24 (50.0)	12 (46.2)	12 (54.5)	0.772
	≥ 24.0 kg/m^2	24 (50.0)	14 (53.8)	10 (45.5)	
eGFR, number (%)					
	< 60.0 mL/min/1.73m^2	32 (66.7)	16 (61.5)	16 (72.7)	0.542
	≥ 60.0 mL/min/1.73m^2	16 (33.3)	10 (38.5)	6 (27.3)	
NT-proBNP, number (%)					
(median)	< 1353 pg/mL	15 (50.0)	9 (47.4)	6 (54.5)	1.000
	≥ 1353 pg/mL	15 (50.0)	10 (52.6)	5 (45.5)	
Atrial fibrillation, number (%)					
	Yes	18 (37.5)	11 (42.3)	7 (31.8)	0.555
	No	30 (62.5)	15 (57.7)	15 (68.2)	
Diabetes mellitus, number (%)					
	Yes	16 (33.3)	5 (19.2)	11 (50.0)	0.034
	No	32 (66.7)	21 (80.8)	11 (50.0)	
Hypertension, number (%)					
	Yes	27 (56.2)	13 (50.0)	14 (63.6)	0.393
	No	21 (43.8)	13 (50.0)	8 (36.4)	
β-blocker use, number (%)					
	Yes	34 (70.8)	19 (73.1)	15 (68.2)	0.758
	No	14 (29.2)	7 (26.9)	7 (31.8)	
LVEF, number (%)					
	< 40%	31 (64.6)	15 (57.7)	16 (72.7)	0.535
	40-49%	7 (14.6)	4 (15.4)	3 (13.6)	

	≥ 50%	10 (20.8)	7 (26.9)	3 (13.6)	
E/e', number (%)					
	< 14	29 (60.4)	16 (61.5)	13 (59.1)	1.000
	≥ 14	19 (39.6)	10 (38.5)	9 (40.9)	
Cardiac index, number (%)					
	< 2.2 mL/m^2	25 (52.1)	14 (53.8)	11 (50.0)	1.000
	≥ 2.2 mL/m^2	23 (47.9)	12 (46.2)	11 (50.0)	
Mean PCWP, number (%)					
	< 15 mmHg	24 (50.0)	12 (46.2)	12 (54.5)	0.772
	≥ 15 mmHg	24 (50.0)	14 (53.8)	10 (45.5)	
Mean PAP, number (%)					
	≤ 20 mmHg	18 (37.5)	8 (30.8)	10 (45.5)	0.375
	> 20 mmHg	30 (62.5)	18 (69.2)	12 (54.5)	
PH, number (%)					
	Yes (Ipc-PH or Cpc-PH)	23 (50.0)	14 (58.3)	9 (40.9)	0.376
	No	23 (50.0)	10 (41.7)	13 (59.1)	

Abbreviations: PAP, pulmonary artery pressure; S2, second heart sound; BMI, eGFR, estimated glomerular filtration rate; NT-proBNP, N-terminal pro-B-type natriuretic peptide; body mass index; LVEF, left ventricular ejection fraction; PCWP, pulmonary capillary wedge pressure; Ipc, isolated post-capillary; Cpc, combined post- and pre-capillary; PH, pulmonary hypertension.

Table S5. Comparison of background characteristics with S2 Width response to exercise-induced PCWP change

		Overall	S2 Width response		*P* value
			**Decreasing (n = 28)**	**Increasing (n = 20)**	
Age, number (%) (median)	< 69 years	23 (47.9)	12 (42.9)	11 (55.0)	0.559
	≥ 69 years	25 (52.1)	16 (57.1)	9 (45.0)	
BMI, number (%) (median)	< 24.0 kg/m^2	24 (50.0)	14 (50.0)	10 (50.0)	1.000
	≥ 24.0 kg/m^2	24 (50.0)	14 (50.0)	10 (50.0)	
eGFR, number (%)	< 60.0 mL/min/1.73m^2	31 (64.6)	17 (60.7)	14 (70.0)	0.555
	≥ 60.0 mL/min/1.73m^2	17 (35.4)	11 (39.3)	6 (30.0)	
NT-proBNP, number (%) (median)	< 1353 pg/mL	15 (51.7)	11 (55.0)	4 (44.4)	0.700
	≥ 1353 pg/mL	14 (48.3)	9 (45.0)	5 (55.6)	
Atrial fibrillation, number (%)	Yes	19 (39.6)	11 (39.3)	8 (40.0)	1.000
	No	29 (60.4)	17 (60.7)	12 (60.0)	
Diabetes mellitus, number (%)	Yes	16 (33.3)	8 (28.6)	8 (40.0)	0.537
	No	32 (66.7)	20 (71.4)	12 (60.0)	
Hypertension, number (%)	Yes	28 (58.3)	15 (53.6)	13 (65.0)	0.555
	No	20 (41.7)	13 (46.4)	7 (35.0)	
β-blocker use, number (%)	Yes	33 (68.8)	19 (67.9)	14 (70.0)	1.000
	No	15 (31.2)	9 (32.1)	6 (30.0)	
LVEF, number (%)	< 40%	31 (64.6)	18 (64.3)	13 (65.0)	0.571
	40-49%	7 (14.6)	3 (10.7)	4 (20.0)	

	≥ 50%	10 (20.8)	7 (25.0)	3 (15.0)	
E/e’, number (%)					
	< 14	30 (62.5)	18 (64.3)	12 (60)	0.772
	≥ 14	18 (37.5)	10 (35.7)	8 (40)	
Cardiac index, number (%)					
	< 2.2 mL/m^2	26 (54.2)	16 (57.1)	10 (50.0)	0.770
	≥ 2.2 mL/m^2	22 (45.8)	12 (42.9)	10 (50.0)	
Mean PCWP, number (%)					
	< 15 mmHg	25 (52.1)	13 (46.4)	12 (60.0)	0.394
	≥ 15 mmHg	23 (47.9)	15 (53.6)	8 (40.0)	
Mean PAP, number (%)					
	≤ 20 mmHg	18 (38.3)	11 (40.7)	7 (35)	0.767
	> 20 mmHg	29 (61.7)	16 (59.3)	13 (65)	
PH, number (%)					
	Yes (Ipc-PH or Cpc-PH)	22 (48.9)	14 (53.8)	8 (42.1)	0.550
	No	23 (51.1)	12 (46.2)	11 (57.9)	

Abbreviations: PCWP, pulmonary capillary wedge pressure; S2, second heart sound; BMI, body mass index; eGFR, estimated glomerular filtration rate; NT-proBNP, N-terminal pro-B-type natriuretic peptide; body mass index; LVEF, left ventricular ejection fraction; PAP, pulmonary artery pressure; Ipc, isolated post-capillary; Cpc, combined post- and pre-capillary; PH, pulmonary hypertension.
